# The Aldehyde Dehydrogenase Superfamily in *Brassica napus* L.: Genome-Wide Identification and Expression Analysis Under Low-Temperature Conditions

**DOI:** 10.3390/ijms26052373

**Published:** 2025-03-06

**Authors:** Ting Jin, Chunhua Wu, Zhen Huang, Xingguo Zhang, Shimeng Li, Chao Ding, Weihua Long

**Affiliations:** 1 College of Rural Revitalization, Jiangsu Open University, Nanjing 210036, China; jinting@jsou.edu.cn (T.J.); dingchao@jsou.edu.cn (C.D.); 2 College of Agronomy, Nanjing Agricultural University, Nanjing 211800, China; 2018201048@njau.edu.cn; 3 College of Agronomy, Northwest A&F University, Xianyang 712100, China; huang_zhen.8@163.com; 4 College of Agronomy, Henan Agricultural University, Zhengzhou 450046, China; xingguozhang@henau.edu.cn; 5 Institute of Agriculture, Tibet Academy of Agriculture and Animal Husbandry Sciences, Lhasa 850032, China; li_shimeng90@163.com

**Keywords:** aldehyde dehydrogenase, *Brassica napus* L., cold stress, expression analysis, natural variation

## Abstract

The Aldehyde Dehydrogenase (ALDH) superfamily comprises a group of NAD^+^ or NADP^+^-dependent enzymes that play essential roles in responding to abiotic stresses in plants. In *Brassica napus* L., however, the increasing frequency of extremely low temperatures during winter in recent years has significantly affected both yield and quality. This study conducted a genome-wide screening of *ALDH* superfamily genes, analyzing their gene structures, evolutionary relationships, protein physicochemical properties, and expression patterns under low-temperature stress to explore the function of the *ALDH* superfamily gene in cold tolerance in *Brassica napus* L. A total of six *BnALDH* genes with significant differences in expression levels were verified utilizing quantitative real-time polymerase chain reaction (qRT-PCR), revealing that *BnALDH11A2*, *BnALDH7B2*, *BnALDH3F5*, *BnALDH12A3*, *BnALDH2B6*, and *BnALDH7B3* all exhibited higher expression in cold-tolerant material 24W233 compared with cold-sensitive material 24W259. Additionally, a single nucleotide polymorphism (SNP) in the *BnALDH11A2* promoter region shows differences between the cold-tolerant (24W233) and the cold-sensitive (24W259) *Brassica napus* varieties, and it may be associated with the cold tolerance of these two varieties. This comprehensive analysis offers valuable insights into the role of *ALDH* family genes in low-temperature stress adaptation in *Brassica napus* and offers genetic resources for the development of novel cold-tolerant cultivars.

## 1. Introduction

Aldehydes are highly reactive compounds that are widely present in plants. They are essential for numerous physiological and metabolic functions, such as the breakdown and synthesis of amino acids, proteins, and carbohydrates [1,2,3,4]. When plants are subjected to various abiotic stresses such as drought, high salt concentrations, and high temperatures, they produce a large amount of aldehyde substances through non-enzymatic peroxidation of the plasma membrane [5,6,7]. While aldehydes are essential during the growth stage, their excessive accumulation can lead to interactions with nucleic acids and proteins, damaging their normal structure and function. This may cause toxic effects on cells and adversely impact plant growth. Thus, it is vital for plants to effectively clear aldehydes to maintain them at a moderate level, preserving normal physiological functions [7,8,9,10]. To mitigate the potential toxic effect caused by the exceeding amount of aldehydes, plants induce the expression levels of aldehyde dehydrogenases (ALDHs) genes [10]. ALDH enzymes can transform both exogenous and endogenous aldehydes into their corresponding carboxylic acids. This process alleviates the toxic effects of aldehydes [7,10] and helps maintain homeostasis to adapt to environmental changes. The ALDH superfamily has already been described and summarized in various crops, including maize, *Arabidopsis*, and rice [11,12,13,14,15]. Eukaryotic ALDHs are divided into 24 protein families (ALDH1-ALDH24) [15,16], with 14 plant-specific subfamilies (ALDH2, ALDH3, ALDH5-7, ALDH10-12, ALDH18-24) [15]. The majority of amino acid sequences encoded by ALDH members contain three conserved regions: (1) cysteine (PS00070) active sites; (2) glutamate (PS00687) active sites; and (3) a Rossmann fold, specifically the GxGxxG coenzyme binding site [10]. These enzymes perform four primary mechanisms in living organisms: detoxification, metabolism, osmoprotection, and NAD(P)H regeneration [7,8,9,10].

In recent years, extensive research has confirmed that ALDHs also play a pivotal role in regulating abiotic stress responses, in addition to their involvement in plant growth regulation [17]. *ALDH* genes predominantly function by eliminating reactive oxygen species (ROS) and excess aldehydes in response to abiotic stresses [5,7,18]. Upon receiving stress signaling, the overproduction of ROS and the activation of the *ALDH* gene promoters occur simultaneously. This induces *ALDH* gene expression, leading to a substantial accumulation of ALDH proteins. Plant stress tolerance is enhanced both by transforming toxic aldehydes into carboxylic acids and by decreasing lipid peroxidation [7,18,19,20,21]. These findings highlight the importance of ALDHs in maintaining environmental adaptation. In *Arabidopsis*, *AtALDH3* is upregulated under certain conditions, such as ABA, salinity, and drought stress. Transgenic *Arabidopsis* lines with overexpression of the *AtALDH3* gene exhibited lower levels of aldehydes produced by membrane lipid peroxidation compared with wild-type plants, suggesting that *AtALDH3* may prevent aldehydes accumulation through detoxification, thereby improving drought and salt stress tolerance [22]. Heterologous overexpression of *ZmALDH* from maize-enhanced aluminum tolerance in *Arabidopsis* was achieved by enhancing the ascorbate–glutathione cycle, increasing antioxidant enzyme activity and gene expression, and reducing malondialdehyde levels while promoting hydroxyproline synthesis [23]. Impaired function of *ALDH3I1* and *ALDH7B4* leads to a decline in NAD(P)H concentration and the NAD(P)H/NAD(P) ratio, as well as disrupted glutathione pool regulation. In the *ALDH*-double mutant *Arabidopsis* lines, glucose-6-phosphate dehydrogenase activity increases, while photosynthetic capacity decreases compared to wild-type plants. It indicates that ALDH maintains metabolic balance through sustaining cellular redox balance [24]. In rice, the *OsALDH7* gene regulates aldehyde levels in seeds during drying, thus functioning as the key mechanism of maintaining seed viability [25]. *ScALDH21*-overexpressing transgenic cotton exhibited improved ROS clearance and reduced osmotic stress under drought conditions. Additionally, hydroxyproline accumulation increased by approximately 11.8–304%, leading to significant improvements in salt and drought tolerance [26,27]. In pepper (*Capsicum annuum* L.), 27 *ALDH* genes can be identified, with the *CaALDH11A1* gene expressing upregulation under cold stress [28].

The development of sequencing technologies has led to the identification of more genome sequence variations, and research on the influence of natural variations in promoter sequences on stress tolerance has gained momentum [29,30,31,32,33]. In *Arabidopsis*, based on genome-wide association studies, several significant SNPs were identified within the *AtMATE* gene region. Further research revealed that an 8.5 kb insertion in its promoter acts as a retrotransposon, inhibiting the expression of the *AtMATE* gene and thereby influencing aluminum tolerance [30]. In wheat breeding in China, InDel variations in the *TaPIF4* promoter, introduced through genotypic germplasm collections from Europe, downregulate *TaPIF4* expression under heat stress, affecting the wheat’s heat tolerance [29]. The salt tolerance gene *GmSALT3* exhibits a natural variation characterized by a 3.8 kb retrotransposon insertion within its coding region. This insertion leads to premature transcriptional termination of *GmSALT3*, resulting in the loss of salt tolerance [31,32]. Two novel natural variations were identified in the promoter region of *GmCHX1*, which modulates salt-induced expression. [33]. Additionally, a natural variation was found in the SNP of the promoter region of *ZmHAK11*. SNP-1781C lines exhibited increased *ZmHAK11* transcription under salt stress, promoting Na^+^ efflux from the root xylem and reducing Na^+^ accumulation in aerial parts. In contrast, lines with SNP-1781G exhibited suppressed transcription under salt stress, resulting in higher Na^+^ concentrations and increased salt sensitivity [34].

Using RNA-seq technology to identify candidate genes related to abiotic stress resistance, there are currently multiple transcriptome assembly methods available, with two commonly used approaches being de novo assembly and reference genome-based assembly [35]. De novo assembly does not rely on a reference genome, thereby circumventing potential biases or inaccuracies that may arise from an incomplete or skewed reference genome. This method is particularly suitable for research involving novel species or those with unknown genomes. On the other hand, reference genome-based assembly depends on an existing reference genome and can expedite the analysis process by utilizing detailed gene annotation information. However, its limitation lies in its dependence on the current annotations available [35,36,37,38,39]. Using RNA sequencing technology, transcriptome analysis was conducted in *Brassica napus* to investigate the expression patterns of genes associated with various stress responses [40,41]. Through RNA-seq analysis of two parental materials after low-temperature vernalization treatment, a large number of differentially expressed genes were identified, revealing the regulatory mechanisms of flowering time to enhance cold resistance [42].

*Brassica napus* L., an essential oilseed crop that contributes to the production of vegetables, flowers, honey, forage, and fertilizers [43,44]. Winter-type *Brassica napus* L. is recognized for its high yield, excellent quality, and significant lodging resistance. However, its poor cold tolerance makes winter protection a primary concern in temperate monsoon climates [45,46]. Thus, improving cold tolerance in winter-type *Brassica napus* L. and breeding superior varieties with high endurance to cold stress is critically important [47].

Although ALDHs in other plants have been widely studied, the research focuses on their family members, and their capacity to respond to cold stress in *Brassica napus* L. remains poorly understood. This study identified and analyzed 45 *ALDH* genes within the genome of *Brassica napus* L. The collinearity, phylogeny, and gene structure characteristics were studied. The expression patterns of these genes in various patterns, as well as their responses, were also included in this research. The main cold-responsive gene was selected, and its low-temperature responsive characteristics were explored. Also, this study discusses the contribution of *ALDH* gene natural variations to cold stress responses. This study aims to provide further research on the novel molecular mechanisms through which *Brassica napus* aldehyde dehydrogenases are involved in low-temperature responses, as well as to develop potential genetic markers for improving cold tolerance in *Brassica napus*.

## 2. Results

### 2.1. Identification and Characterization of the ALDH Superfamily of Brassica napus L.

**Figure 1:**
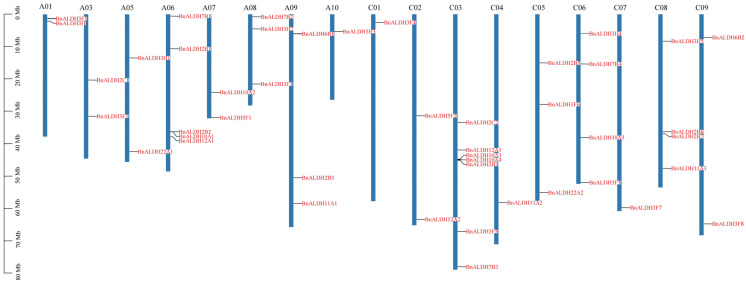
Chromosomal distribution of the *Brassica napus Aldehyde Dehydrogenase* (*ALDH*) superfamily members. Chromosomal mapping was performed using TBtools software based on the physical location information of *ALDH* family members on the chromosomes. The scale on the left indicates the length of the *Brassica napus* chromosomes. The chromosome names are labeled at the top. The physical locations of *BnALDH* genes are marked with short lines.

The ALDH members in *Brassica napus* L. were identified using the Basic Local Alignment Search Tool (BLAST) v2.2.9 and HMMER v3.4. The existence of conserved ALDH domains (PF00171) was verified using the Pfam and NCBI conserved domains database. The candidate *ALDH* members were obtained from the *Brassica napus* (ZS11) genome. To confirm the presence of conserved structural domains, all putative members were analyzed using the SMAT database (http://smart.embl-heidelberg.de/) (accessed on 3 December 2023). Finally, 45 *Brassica napus* L. *ALDHs* (*BnALDHs*) were identified and named according to the AGNC guidelines (Figure 1 and Table S1).

The lengths of BnALDHs range from 114 amino acids to 596, with the molecular weight ranging from 12567.94 (kDa) to 66100.27 (kDa), and the predicted isoelectric points ranging from 5.28 to 9.64. Subcellular localization predictions indicated that 29 out of 45 BnALDHs are localized in chloroplasts, 10 in mitochondria, and only two in the cytoplasm. Signal peptide predictions revealed that most BnALDH members lack signal peptides. Transmembrane domain (TMD) prediction suggests that the primary of the BnALDHs members do not contain TMDs (Table S1).

### 2.2. Distribution and Phylogenetic Analysis of the ALDH Superfamily in Brassica napus L., Arabidopsis, and Soybean

The physical locations of all *ALDH* genes in *Brassica napus* L. (rapeseed) on the chromosomes were determined and mapped using TBtools software (Figure 1). The results indicate that *ALDH* genes are distributed across most chromosomes, with the exception of A02 and A04. Among these, A03 contains the highest number of *ALDH* genes (7), while A10, C01, C04, and C07 each contain only one gene. Other chromosomes contain between two and five members (Figure 1).

**Figure 2:**
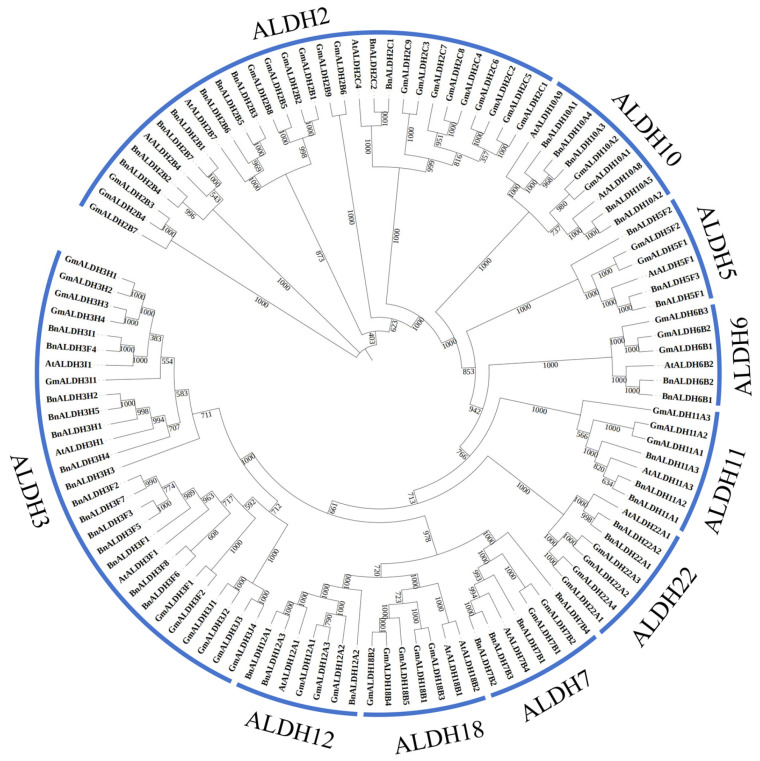
Phylogenetic analysis of multi-species ALDH members. A total of 114 ALDH proteins were identified from *Glycine max*, *Arabidopsis thaliana*, and *Brassica napus*. These proteins were aligned with ClustalX2.0, and a phylogenetic tree was generated using the neighbor-joining (NJ) method in MEGA6. The labels outside the circle indicate the names of the ALDH subfamilies, while the values on the branch nodes represent the bootstrap values (in percentage).

Phylogenetic relationships of the ALDH superfamily in *Brassica napus* L. were analyzed by performing a multiple sequence alignment of ALDH proteins from soybean, *Arabidopsis*, and *Brassica napus* L. (Figure 2) using MEGA v6.0 software. The analysis revealed that the ALDH superfamily can be divided into 10 subfamilies. Soybean contains the largest number of ALDH members (53), while *Arabidopsis* has the fewest (16). Both soybean and *Arabidopsis* have ALDH superfamilies composed of 10 subfamilies. The phylogenetic trees showed that the 45 BnALDH are divided into 9 subfamilies: ALDH2, ALDH3, ALDH5, ALDH6, ALDH7, ALDH10, ALDH11, ALDH12, and ALDH22. However, no ALDH18 family was identified in *Brassica napus* L. In *Brassica napus* L., the subfamilies vary in size, with the ALDH3 subfamily containing the most members (14) and the ALDH6 and ALDH22 subfamilies containing the fewest (2) (Figure 2).

### 2.3. Conserved Motifs and Gene Structure Analysis in the ALDH Superfamily of Brassica napus L.

**Figure 3:**
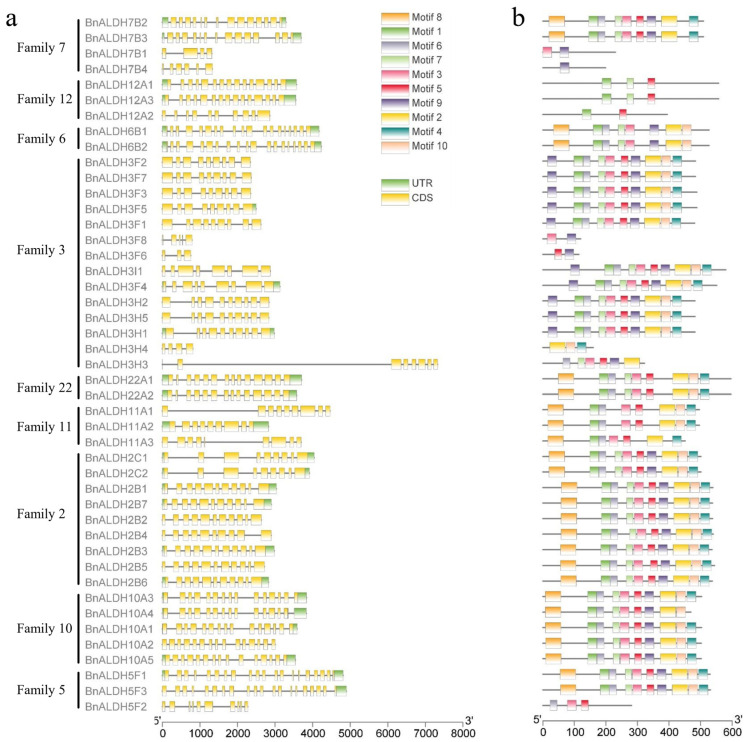
Analysis of exon–intron structures and conserved motifs of *Brassica napus* ALDH superfamily members. (**a**) The structure of exon–intron structures of the *BnALDH* genes. The green boxes represent untranslated (UTR) regions, yellow boxes represent exons, and gray lines represent introns. (**b**) Shown are the top ten motifs based on motif E-value. Each motif is marked by a box of a distinct color.

Gene structure analysis of the ALDH family in *Brassica napus* L. revealed that *BnALDHs* contain 3 to 20 introns and 2 to 19 exons (Figure 3a). The ALDH6, ALDH11, and ALDH22 subfamilies contain 19, 9, and 14 introns, respectively, indicating a highly conserved gene structure. Other subfamilies, such as ALDH2 and ALDH10, exhibit relatively consistent intron numbers, whereas ALDH3, ALDH5, ALDH7, and ALDH12 show greater variation in gene structure, with significant differences in intron-exon organization.

Conserved motif analysis of the amino acid sequences of BnALDHs was performed using MEME (version 5.5.7, http://meme-suite.org/tools/meme) (accessed on 12 December 2023). A total of 10 conserved motifs (motifs 1–10) were identified across all BnALDH proteins. Some subfamilies, such as ALDH2, ALDH6, ALDH10, and ALDH22, exhibit highly conserved motifs, which may indicate functional similarities. Other subfamilies, such as ALDH5, ALDH7, ALDH11, and ALDH12, share at least one motif, although their motif profiles differ significantly. The most common motifs are motif 3 and motif 5, present in 39 of the 45 BnALDHs (86.67%). In contrast, motif 8 is relatively rare, appearing in only 25 BnALDHs (55.56%). Certain motifs are absent in specific subfamilies. For example, motif 8 is absent in ALDH3, motifs 4 and 5 are missing in ALDH6, and ALDH11 lacks motifs 7 and 9. ALDH22 does not contain motif 9, and ALDH12 lacks several motifs, retaining only motifs 1, 5, and 7. Notably, motif 9 appears twice in some members (Figure 3b).

### 2.4. Genetic Collinearity of ALDH Genes in Brassica napus L.

**Figure 4:**
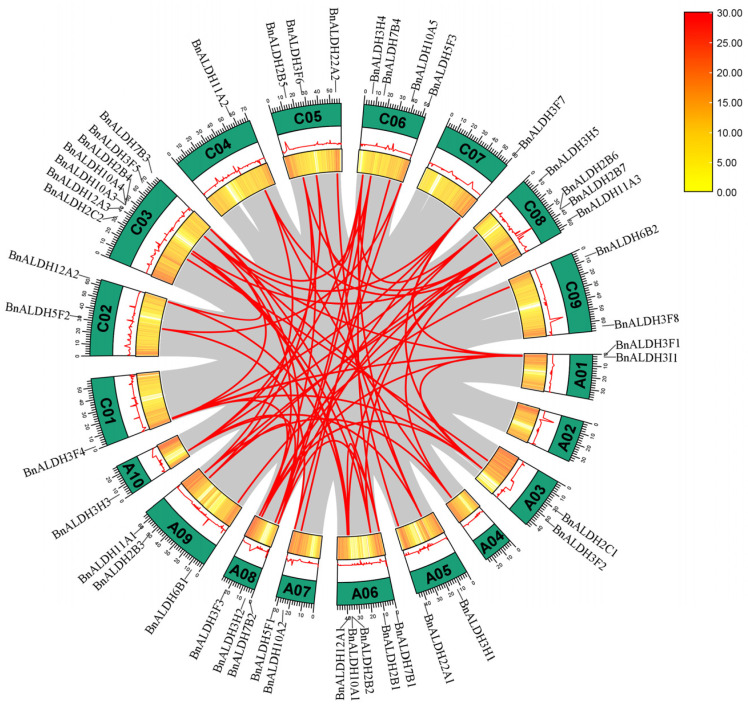
Expansion patterns of *Brassica napus ALDH* genes. Intraspecific collinearity among the 45 members of the *BnALDH* family was analyzed and visualized using the advanced Circos module in TBtools. The different colors of the lines represent the gene pairs of 45 genes in the *ALDH* gene family of *Brassica napus*. The genome-wide gene density, the distribution of GC content, and the chromosomes are demonstrated by rings from the inside to the outside, respectively.

Gene family expansion can occur through tandem duplication and segmental duplication. To investigate the expansion of the *ALDH* family in *Brassica napus* L., the physical locations of the genes were analyzed using TBtools software. Tandem duplication genes and genes located in large segmental duplication regions were identified. The results revealed two pairs of tandem duplication genes: *BnALDH10A3*/*BnALDH10A4* on chromosome C03 and *BnALDH2B6*/*BnALDH2B7* on chromosome C08. Additionally, 43 segmental duplications were detected among the 45 *BnALDH* genes (Figure 4). These findings demonstrate that tandem and segmental duplications are key drivers of the *ALDH* gene family’s expansion.

### 2.5. Tissue Expression Analysis of the ALDH Family in Brassica napus L.

**Figure 5:**
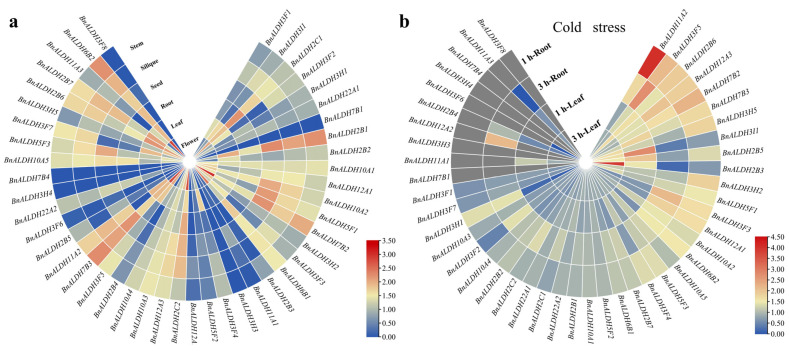
Tissue expression profiles and cold induced expression profiles of *BnALDHs*. (**a**) The tissue expression heat map of *BnALDHs* was constructed based on log_10_^(TPM+1)^ values. The TPM values were obtained from BnIR database (ZS11 library). The color scale is used to indicate the relative transcript abundance of the *BnALDHs* in various plant organs, including stems, buds, roots, leaves, flowers, seeds, siliques. (**b**) Heap map of cold induced expression profiles in root tissues of *ALDHs* in *Brassica napus* which was subjected to cold stress for 1 h and 3 h. Heatmaps were generated using TBtools v2.154 software. A heat map was generated using the log_2_ ratio of the FPKM value under cold stress to the FPKM value of the control for 45 *BnALDHs*.

RNA sequencing data (Transcripts Per Million, TPM) from seven tissues—flowers, leaves, roots, seeds, siliques, and stems—were collected from the *Brassica napus* multi-omics information resource (BnIR) (ZS11 library) to analyze the expression profiles of *BnALDHs* and investigate their potential roles (Figure 5a). Certain *BnALDHs*, such as *BnALDH10A2*, *BnALDH5F1*, *BnALDH11A1*, and *BnALDH5F3*, displayed the highest levels of expression in seeds, indicating potential roles in seed development. In contrast, other *BnALDHs*, including *BnALDH2B1*, *BnALDH7B2*, *BnALDH2C2*, *BnALDH3F5*, *BnALDH7B3*, *BnALDH2B6*, and *BnALDH2B7*, showed relatively high expression levels across multiple tissues, suggesting broader functional roles. Additionally, a few *BnALDHs* (e.g., *BnALDH7B1*, *BnALDH7B4*, *BnALDH3H3*, *BnALDH3H4*, *BnALDH3F6*, and *BnALDH3F8*) showed no detectable expression in any of the analyzed tissues. These differential expression patterns suggest that *BnALDHs* may play tissue-specific roles during the development of *Brassica napus* L.

### 2.6. Expression Pattern of BnALDHs in Response to Cold Stress

The response of *BnALDHs* to cold stress was investigated using transcriptome data from the BnIR. After 1 h of cold stress, several *BnALDHs*, including *BnALDH11A2*, *BnALDH7B2*, and *BnALDH3F5*, showed significant upregulation. By 3 h of cold stress, additional *BnALDH* genes exhibited altered expression levels. Root tissues demonstrated higher sensitivity to cold stress compared to leaves. Specifically, *BnALDHs* such as *BnALDH2B6*, *BnALDH12A3*, *BnALDH7B3*, *BnALDH3H2*, and *BnALDH5F1* were upregulated in both leaf and root tissues, while some genes displayed tissue-specific responses: 4 genes were upregulated only in leaves, and 13 were upregulated only in roots. Among all genes, *BnALDH11A2* showed the highest upregulation rate. In contrast, *BnALDH3I1*, *BnALDH2B3*, *BnALDH3H1*, *BnALDH3F6*, and *BnALDH11A3* were downregulated during the initial period of stress. After 3 h of cold stress, *BnALDH3H1* remained downregulated, while other genes exhibited stable expression levels that were not significantly affected by the cold stress (Figure 5b). 

**Figure 6:**
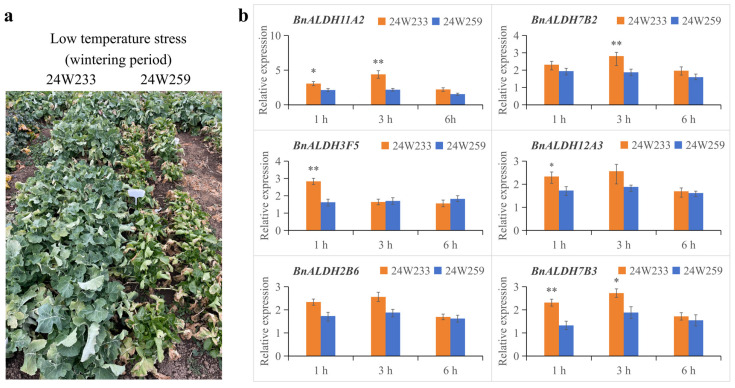
Expression pattern analysis of *BnALDHs* in response to cold stress. (**a**) Field phenotypes of cold-resistant *Brassica napus* variety 24W233 and cold-sensitive variety *Brassica napus* 24W259 under cold stress during the overwintering period. There was a low-temperature (the daily average temperature below 0 °C) period for nine days in December 2023, which formed a natural assessment environment. The phenotypes of candidate lines were detected on the third day after that period. (**b**) Relative expression levels of six *BnALDHs* in 24W233 and 24W259 in response to cold stress. *Brassica napus* seedlings were treated with 4 °C or 22 °C for 1 h, 3 h, and 6 h. The normal temperature group at each time point was used as the control. Three biological replicates were performed, with *n* = 3 × 3 = 9. The data are shown as mean values accompanied by standard deviation (SD). The statistical significance was assessed by means of a two-sided Student’s *t*-test, with significance levels indicated as * *p* < 0.05 and ** *p* < 0.01.

In a previous field survey on cold tolerance traits, the cold-tolerant *Brassica napus* variety 24W233 and the cold-sensitive variety 24W259 were identified. At the overwintering stage, when the plants reached the 7-leaf stage, 24W233 exhibited more vigorous growth compared to 24W259 (Figure 6a). To further analyze cold stress responses, 3-week-old plants of 24W233 and 24W259 were subjected to cold stress treatment at 4 °C in a controlled environment. Mixed samples of leaves and roots (1:1 ratio) were collected after 1 h, 3 h, and 6 h of cold stress treatment. To explore the expression patterns of several *BnALDH* genes, including *BnALDH11A2*, *BnALDH7B2*, *BnALDH3F5*, *BnALDH12A3*, *BnALDH2B6*, and *BnALDH7B3*, quantitative real-time polymerase chain reaction (qRT-PCR) analysis was carried out. All genes were upregulated in both varieties of *Brassica napus* L. Interestingly, after 1 and 3 h of cold stress, the cold-tolerant variety 24W233 exhibited a higher upregulation rate compared to the cold-sensitive variety 24W259. However, after 6 h of cold stress, there were no significant differences in the expression levels of these six genes between the two varieties, suggesting that *BnALDHs* primarily play a crucial role in the early stages of the cold stress response in *Brassica napus* L.

### 2.7. Promoter Variations and Salt Tolerance of BnALDH11A2

**Figure 7:**
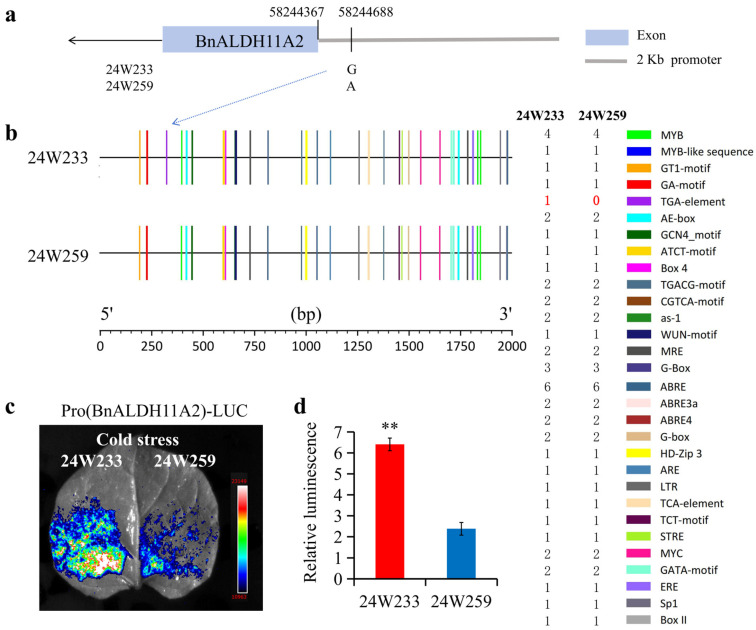
Activity of different *BnALDH11A2* promoters in response to cold stress. (**a**) The diagram shows the natural variation site in the *BnALDH11A2* promoter and its two corresponding haplotypes 24W233(G) and 24W259(A). (**b**) Analysis of cis-acting elements of two haplotype promoters, 24W233 (G) and 24W259(A). (**c**) Detection of promoter activity under cold stress of two haplotype promoters. After treatment at 4 °C for 45 min, the activities of the two haplotype promoters were transiently expressed. Each haplotype promoter drove the LUC reporter gene. Images were captured with an in vivo plant imaging system. (**d**) Quantification of luminescence intensity shown in (**c**). The data values represent the mean of three biological replicates. The data values correspond to the mean of three biological replicates. Each of these biological replicates comprises three technical replicates. The error bars denote the standard deviation across the three biological replicates. Statistical significance was assessed using Student’s *t*-test, where ** indicates *p* < 0.01.

Transcriptome data and qRT-PCR results demonstrated that *BnALDH11A2* is significantly upregulated in response to cold stress, with the highest level of upregulation observed after 1 h (Figure 5b and Figure 6). To investigate the potential causes of these expression patterns, we sequenced and aligned the promoter and coding regions of *BnALDH11A2* (C04_58241738–58246561) in cold-tolerant 24W233 and cold-sensitive 24W259 lines. The results revealed a single-nucleotide polymorphism (SNP) at position C04_58244688, with G in 24W233 and A in 24W259 (Figure 7a). We forecasted cis-acting elements within the promoter region (located 2 kb upstream of the transcription start site) of *BnALDH11A2*. Several cis-acting elements associated with growth, light responses, stress, and hormone were detected (Table S2 and Figure 7b). Among these, multiple stress-responsive elements were found, such as the antioxidant response element (ARE) associated with anaerobic induction, the metal response element (MRE) related to oxidative stress, and the MYB element linked to drought, cold, or salt stress. These findings imply that BnALDH11A2 might be involved in responding to diverse abiotic stresses, such as cold stress. Notably, we identified a TGA element in the promoter region of 24W233, but no such element was found in 24W259. This variation is attributed to the SNP at position C04_58244688, resulting in changes to the cis-acting elements in the promoter region (Figure 7b).

The promoters of *BnALDH11A2* from both 24W233 and 24W259 were cloned to construct dual-luciferase reporter vectors. The activity of these promoters under cold stress was examined in *Nicotiana benthamiana*. Intravital imaging demonstrated that the LUC signal in Pro(BnALDH11A2^24W233^)-LUC was significantly stronger compared with Pro(BnALDH11A2^24W259^)-LUC, consistent with the results of the dual luciferase activity assay (Figure 7d). These results suggest that the promoter of *BnALDH11A2* from the cold-tolerant species 24W233 is more responsive to cold stress than the promoter from the cold-sensitive species 24W259.

## 3. Discussion

ALDH serves as an essential aldehyde scavenger in plants, playing a vital role in mitigating the effect of adversity stress through clearing aldehydes and maintaining cellular homeostasis [7,10]. In this study, a total of 45 members from the *ALDH* superfamily (Figure 1) were identified during the genome-wide analysis of *Brassica napus* L. In comparison, the number of family members in *Arabidopsis* and soybean is 16 and 53, respectively. The variation in the number of ALDH members among different species may be influenced by genome size and evolutionary factors [48]. Other research has categorized eukaryotic ALDHs into 24 protein families, with 14 identified in plants and 10 in the primary angiosperm lineage [15]. Phylogenetic analysis reveals that the ALDHs in *Brassica napus* L. can be classified into nine distinct families, namely ALDH3, ALDH12, ALDH7, ALDH22, ALDH11, ALDH6, ALDH5, ALDH10, and ALDH2 (Figure 2). The absence of members in ALDH18 is likely attributed to various degrees of gene loss within the ALDH gene family during evolution. The highest numbers of members are observed in BnALDH2 and BnALDH3 families, which together account for 50% of the ALDH family members in *Brassica napus* L. This phenomenon is also observed in other species [23,27,48], highlighting the evolutionary conservation and essential functions of these families. The variation in the number of introns and exons is primarily caused by gene segment fusion or rearrangement [49]. Gene structure analysis of 45 *ALDH* family members in *Brassica napus* L. reveals that a similar intron–exon structure is present within members from various families, including *ALDH12*, *ALDH6*, *ALDH3*, *ALDH22*, *ALDH2*, *ALDH10*, and *ALDH5*. This is potentially due to gene replication events (Figure 4), which lead to both an increase in gene number and functional diversification of *ALDH* family genes [23,27,48]. Meanwhile, different families exhibit distinct gene structures, reflecting significant variation across families.

The participation of the *ALDH* gene in responses to abiotic stress occurred through multiple mechanisms, including signaling pathways regulation, transcription factor activity, ROS scavenging, and protein interaction in higher plants. In this study, the expression patterns of *BnALDH* genes were analyzed with the aim of obtaining a better understanding of the function of the *ALDH* gene in the responses to low-temperature stress in *Brassica napus* L. A significant number of *BnALDH* genes were found to be induced upon exposure to cold stress. To explore this further, this study selected one sample of cold-tolerant *Brassica napus* L. 24W233 and one sample of cold-sensitive material 24W259 (Figure 6a). By examining the six genes that showed remarkable upregulation in transcriptomes under cold stress, we performed a qRT-PCR assay, which revealed consistent expression patterns between *BnALDH* genes and the public transcriptome data. The results show that the following genes were significantly upregulated under cold stress in cold-tolerant materials but showed no induction in cold-sensitive materials: *BnALDH11A2*, *BnALDH7B2*, *BnALDH3F5*, *BnALDH12A3*, *BnALDH2B6*, and *BnALDH7B3*. This suggests a positive correlation between the induction of *BnALDHs* under cold stress and the cold tolerance of *Brassica napus L*. The ALDH2 family is primarily located in the mitochondria or cytoplasm and plays a central role in the oxidation and detoxification of acetaldehyde [50]. The *ALDH2* gene (rf2) in maize was the first discovered *ALDH* gene. It encodes a mitochondrion protein that participates in the regulation of anther development, marking significant importance in the restoration of male fertility [51]. In rice, OsALDH2B1, which is an ALDH located in the mitochondria, plays a regulatory role in the G protein, salicylic acid, and jasmonic acid signaling pathways. As a result, it has an impact on the growth, development, and immune responses of the plant [52]. In Moso bamboo, PeALDH2B2 interacts with the Gβ protein PeGPB1, responding to drought stress [53]. Heterologous expression of the peanut gene *AhALDH2B6* in soybeans significantly increases ALDH activity in transgenic soybeans while reducing malondialdehyde levels, leading to increased peroxidase and catalase concentrations. Furthermore, several genes associated with oxidoreductases are upregulated, collectively contributing to the cold tolerance of the plant [54]. The *ALDH3* gene family is mainly involved in responses to various adversity stresses, including salt stress, drought stress, heavy metal stress, and abscisic acid stress [55,56]. *AhALDH3H1* maintains cellular homeostasis in transgenic plants under saline-alkali stress by enhancing ALDH activity, reducing malondialdehyde levels, regulating gene expressions associated with cell wall structures, and accelerating metabolite transport through ABC transporters, thereby improving plant tolerance [48]. *ZjALDH3F3* in tobacco participates in the response to heat stress by oxidizing aromatic and aliphatic aldehydes, leading to decreased ROS accumulation and increased antioxidant enzyme activity, while activating genes associated with ABA signaling [56]. ALDH7 is a highly conserved protein in the acetaldehyde dehydrogenase family. In plants, it can be induced by osmotic stress, such as salt and drought [57,58], and it clears toxic aldehydes under stress conditions, reducing ROS production [58]. Overexpression of *ALDH7* in *Arabidopsis* and tobacco significantly enhances resilience to stress by clearing harmful aldehydes produced during redox processes and increasing prooxidant capacity [58]. Overexpression of *ALDH7* in *Arabidopsis* and tobacco significantly enhances resilience to stress by clearing harmful aldehydes produced during redox processes and increasing prooxidant capacity [57]. The ALDH11 gene encodes glyceraldehyde 3-phosphate dehydrogenase, which catalyzes the conversion of NADP^+^ and glyceraldehyde-3-phosphate into NADPH and 3-phosphoglyceric acid. This reaction provides the majority of the NADPH required for mannitol biosynthesis in many plants [59]. The ALDH12 family encodes glutarate semialdehyde dehydrogenase (GSALDH), which converts glutamic semialdehyde (GSAL) into glutamic acid in an NAD-dependent manner. Its expression is induced by exogenous proline and participates in the responses to salt and drought stresses by maintaining proline homeostasis [60]. In summary, *ALDH* genes participate in responses to abiotic stresses through diverse mechanisms in plants. Further research is needed to explore the specific mechanisms of the six genes highlighted in this study.

The cold tolerance of *Brassica napus* lines is evaluated by the freezing injury grades, which are confirmed by the phenotypes of the leaves at the seedling stage in winter [61]. Therefore, we conducted a phenotypic analysis of seedling-stage *Brassica napus* to identify cold tolerance phenotypes and screened for cold-tolerant and cold-sensitive materials (Figure 6a). Research on cold tolerance in *Brassica napus* has predominantly focused on the bud and seedling stages [62,63,64]. Under cold stress, the seedling survival rates of two *Brassica napus* varieties were compared, along with their physiological responses at the seedling stage. It was found that the 1801 variety exhibited a stronger cold stress response capability compared to the C20 variety. The 1801 variety was able to cope with cold stress by maintaining hormone levels, antioxidant activity, and osmotic regulation [63]. Exogenous trehalose significantly enhances cold stress tolerance in *Brassica napus* seedlings by regulating antioxidant activity and osmotic balance, as well as participating in trehalose metabolism and signaling networks [64]. In subsequent studies, the measurement of physiological data, such as fresh weight and seedling height, will serve as a robust supplement to the evaluation of cold tolerance in *Brassica napus*.

The promoter, an essential element that accurately regulates gene expression, determines the directionality and efficiency of transcriptional initiation and controls the timing, spatial distribution, and level of gene expression [65,66]. Natural variations in promoters are associated with phenotypic changes related to stress tolerance [31,32,33,34]. In the previous investigation on cold tolerance in field conditions, two materials, cold-tolerant species 24W233 and cold-sensitive species 24W259, were identified. Both transcriptome data (Figure 5b) and qRT-PCR analysis (Figure 6b) demonstrated that *BnALDH11A2* is upregulated in response to low-temperature induction. Sequencing and cloning of the *BnALDH11A2* gene from both materials reveal that both sequences present natural variations in the promoter region SNP (C04_58244688). These variations result in changes in the cis-acting elements in the promoter regions, with a TGA element forming in the promoter region of 24W233, the cold-tolerant material (Figure 7b). The TGA element plays a crucial role in regulating multiple processes in plants, including growth, hormone responses, and abiotic stress responses [67,68,69,70]. In tobacco, it participates in the auxin signaling pathway, posing an underlying influence on the development of fiber cells [67]. In *Arabidopsis*, it combines with ARF7 and ARF19, regulating the plant height [68]. The TGA element in the promoter of *CbCOR15a* responds to cold stress by mediating auxin signaling, thereby enhancing the adaptive capacity of tobacco under low temperatures [69]. In wheat, the TGA element can combine with auxin response factors (ARFs), regulating the response to auxin. Natural variations in the *TaPYL4-2* allele resulted in the exclusive presence of the TGA element in the promoter of the Hap-2A-1 haplotype. It regulates the transcriptional activity of *TaPYL4-2A*, thereby influencing wheat growth and stress responses [70]. This study identified a G/A allele variation in the promoter of *BnALDH11A2*, resulting in the formation of a TGA element that affects the cold tolerance of the material. The Luciferase reporter assay utilizing the promoter further confirmed that the promoter (G at C04_58244688 with TGA element) in 24W233 is a cold-tolerant haplotype (Figure 7).

## 4. Materials and Methods

### 4.1. Identification and Physicochemical Properties Analysis of the ALDH Superfamily in Brassica napus L.

Genome data and structural annotation information (Brana_ZS11_HZAU_V1.0) of *Brassica napus* L. (Zhongshuang11, ZS11) were obtained from the NCBI database. The *ALDH* gene sequences of *Arabidopsis* were downloaded from the TAIR database. The *ALDH* gene sequences of soybean were obtained from the Phytozome database. The genome of *Brassica napus* L. was aligned using BLAST v2.2.9, with the ALDH protein sequence of *Arabidopsis* as the query. The significance threshold was set as 1e-5 to identify protein sequences of candidate genes. The structural domains of the ALDH superfamily were retrieved from the Pfam database (http://pfam.xfam.org) (accessed on 3 December 2023) using InterPro (https://www.ebi.ac.uk/interpro/) (accessed on 3 December 2023) and NCBI CD-Search (https://www.ncbi.nlm.nih.gov/Structure/bwrpsb/bwrpsb.cgi) (accessed on 3 December 2023) to confirm the presence of only ALDH-conserved structural domains (PF00171). Physicochemical properties, including the number of amino acids, molecular weight, isoelectric point, and hydrophobicity, were predicted for the ALDH superfamily members in *Brassica napus* L. using TBtools v2.152 [71]. Subcellular localization was predicted using Cell-PLoc 2.0 [72].

### 4.2. Phylogenetic Analysis of the ALDH Superfamily

Multiple sequence alignment was performed on the ALDH family protein from *Brassica napus* L., *Arabidopsis* [12], and soybean [48] (Table S3) using MEGA v6.0 [73]. The phylogenetic tree was constructed using the neighbor-joining method [74], with bootstrap values set to 1000. The resulting phylogenetic tree was refined using iTOL (https://itol.embl.de/) (accessed on 9 December 2023) [75].

### 4.3. Conserved Motifs and Gene Structure Analysis 

A total of 45 ALDH protein sequences from Brassica napus L. were analyzed using MEME (https://meme-suite.org/meme/tools/meme) (accessed on 12 December 2023), with the number of motifs set to 10. Gene structure and conserved motifs were visualized using the Gene Structure View (Advanced) module in TBtools v2.152.

### 4.4. Chromosomal Mapping and Collinearity Analysis

Chromosomal mapping was performed using TBtools software based on the physical location information of *ALDH* family members on the chromosomes. The genome annotation file (zs11.v0.gff3) for Brassica napus L. was downloaded from NCBI. Intraspecific collinearity among the 45 members of the *BnALDH* family was analyzed and visualized using the advanced Circos module in TBtools v2.152.

### 4.5. Expression Patterns in Different Tissues Under Cold Stress

Transcriptome data of *ALDH* genes in various tissues and root tissues of *Brassica napus* L. ZS11 material under cold stress were downloaded from the BnTIR online database [76,77] (https://yanglab.hzau.edu.cn/BnIR) (accessed on 21 February 2024). Heatmaps showing the expression patterns of *ALDH* family members in *Brassica napus* L. under cold stress across different tissues were generated using TBtools software.

### 4.6. Assessment of Cold Tolerance in Brassica napus L. at the Seedling Stage

Seeds of *Brassica napus* L. were sown directly in the field in October 2023. There was a low-temperature period (the daily average temperature below 0 °C) for 9 days in December 2023, which formed a natural assessment environment. Moreover, on the third day after this low-temperature period, when the *Brassica napus* L. candidate lines were at the seven-leaf stage, inspections of the seedling phenotypes were carried out and cold injury assessments were conducted, with photographic records taken.

### 4.7. Materials Cultivation, RNA Extraction, and qRT-PCR

Two varieties, 24W233 (cold-tolerant) and 24W259 (cold-sensitive), were selected for this study. Seeds of uniform size and plump grains, free from disease or insect damage, were sterilized with a 1% sodium hypochlorite solution for 15 min, rinsed three times with distilled water, and then dried. Seeds were sown in a sterilized medium consisting of vermiculite and nutrient soil in a 1:1 ratio, with 10 seeds per pot. Seedlings were grown in growth chambers with a 12-h light/12-h dark photoperiod and a temperature regime of 25 °C/20 °C. After thinning to three plants of similar growth potential, the seedlings were grown for three weeks. Samples were collected for DNA extraction. For expression pattern analysis under cold stress, seedlings of similar growth potential were transferred to growth chambers set at 4 °C, with a control group maintained at 22 °C. Samples were collected at 1, 3, and 6 h after treatment for qRT-PCR analysis. After collection, samples were immediately frozen in liquid nitrogen. RNA was extracted using the total RNA extraction kit (TIANGEN, Beijing, China), and cDNA was synthesized by reverse transcription using the PrimeScript RT reagent Kit (Takara, Tokyo, Japan). Fluorescent quantitative primers (Table S4) were designed separately using the NCBI BLAST online tool (https://blast.ncbi.nlm.nih.gov/Blast.cgi?PROGRAM=blastn&PAGE_TYPE=BlastSearch&LINK_LOC=blasthome) (accessed on 5 March 2024) to detect the expression levels of *BnALDH11A2* at different time periods under 4 °C treatment, with the normal temperature group at each time point as the control and *BnActin2* as the internal reference gene [78]. A 20 μL reaction mixture was prepared according to the manufacturer’s instructions for the SuperReal PreMix Plus (SYBR Green) (TIANGEN, Beijing, China), and qRT-PCR was performed using the Roche 480 system (Roche, Basel, Switzerland). Three technical replicates were performed for each sample, and three biological replicates were included in the experiment. Relative expression levels were calculated using the 2^−ΔΔCt^ method [79].

### 4.8. Gene Cloning and Cis-Acting Elements Analysis of BnALDH11A2 Gene

The *BnALDH11A2* genome sequence (C04_58241738..58246561) was retrieved from the *Brassica napus* L. genome ZS11 database (Brana_ZS11_HZAU_V1.0). Specific primers (Table S4) were designed to amplify *BnALDH11A2* using DNA and cDNA from both 24W233 and 24W259 as templates. Amplification was performed using 2 × Taq Plus Master Mix (Vazyme, Nanjing, China), and the resulting products were sequenced (Sangon Biotech, Shanghai, China). The promoter and coding region sequences of the *BnALDH11A2* gene were analyzed for cis-acting elements using PlantCARE online software (http://bioinformatics.psb.ugent.be/webtools/plantcare/html/) (accessed on 17 March 2024) and visualized with CFVisual_V2.1.5 to explore the potential functions of core elements and family members.

### 4.9. Examining Promoter Activity

The promoters of the *BnALDH11A2* gene from the 24W233 and 24W259 materials were cloned into the pGreenII 0800-LUC vector to generate the recombinant plasmids ProBnALDH11A2^24W233^-LUC and ProBnALDH11A2^24W259^-LUC. These plasmids were transformed into *Agrobacterium tumefaciens* GV3101 (pSoupP19) using the freeze-thaw method. After confirming successful transformation by PCR, the bacterial suspension was adjusted to an OD600 of 0.6 and allowed to stand for 30 min. The suspension was then injected into the leaves of *Nicotiana benthamiana* using a 1 mL syringe. The injected leaves were incubated in the dark at room temperature for 16 h, followed by 45 min of light incubation at 4 °C. Subsequently, 1 mmol/L luciferase substrate was sprayed onto the back of the injected leaves, and the leaves were treated in the dark for 5 min at room temperature. LUC activity was observed with in vivo plant imaging system (Tanon, Shanghai, China).

To quantify the fluorescence values (firefly luciferase/renilla luciferase, LUC/REN), the TransDetect Double-Luciferase Reporter Assay Kit (TransGen Biotech, Beijing, China) was used. Three tobacco leaf discs (6-8 mm in diameter) were ground into powder, and 300 μL of 1 × Cell Lysis Buffer was added. The mixture was thoroughly blended and lysed for 5 min at room temperature, followed by centrifugation at 12,000 rpm for 2 min. The supernatant was collected for analysis. For the luciferase assay, 100 μL of Luciferase Reaction Reagent I was equilibrated to room temperature and added to a white opaque 96-well plate. Then, 20 μL of the lysate was carefully transferred into the plate. After mixing by horizontal shaking, the firefly luciferase activity was measured using an enzyme labeling instrument. Subsequently, 100 μL of Luciferase Reaction Reagent II was added to the plate, mixed by horizontal shaking, and the renilla luciferase activity was measured.

## 5. Conclusions

In this study, the *BnALDH* superfamily was screened in the *Brassica napus* L. genome via bioinformatics methods, and 45 of its members were successfully identified. Phylogenetic analysis classified these members into nine distinct families. Multiple gene duplications were observed within the *BnALDH* genes. Most *BnALDH* members exhibited upregulation under cold stress, suggesting their potential roles as molecular markers for assessing cold tolerance in *Brassica napus* L. and as candidate genes for breeding cold-tolerant varieties. Notably, an elite allele variation (G/A) was identified in the promoter region of *BnALDH11A2*, which is associated with enhanced cold tolerance. In this study, we analyzed the relationship between SNPs and cold tolerance in only two varieties and validated the findings using LUC assays. To gain a deeper understanding of the association between this locus and cold tolerance, and to provide foundational materials for molecular breeding of cold-tolerant rapeseed, we plan to expand the sample size in future experiments, with the aim of achieving further significant discoveries in subsequent studies.

Currently, this variation could serve as a valuable haplotype for improving low-temperature resistance in *Brassica napus* L. This study not only enriches our comprehension of the functional roles of the *ALDH* gene family but also offers vital genetic resources for future endeavors aimed at enhancing stress resistance in *Brassica napus* L.

## Data Availability

Data are contained within the article and Supplementary Materials.

## Supplementary Materials

The following supporting information can be downloaded at: https://www.mdpi.com/article/10.3390/ijms26052373/s1.

## Abbreviations

The following abbreviations are used in this manuscript:

ALDH	Aldehyde dehydrogenase
ARE	Antioxidant response element
ARFs	Auxin response factors
BLAST	Basic local alignment search tool
GSAL	Glutamic semialdehyde
GSALDH	Glutarate semialdehyde dehydrogenase
LUC	Luciferase
LUC/REN	Firefly luciferase/renilla luciferase
MRE	Metal response element
NJ	Neighbor-joining
qRT-PCR	Quantitative real-time polymerase chain reaction
ROS	Reactive oxygen species
SNP	Single-nucleotide polymorphism
TMD	Transmembrane domain
ZS11	Zhongshuang11
